# The HPA axis and kynurenine pathway: exploring the role of stress and neuroinflammation in treatment-resistant depression

**DOI:** 10.1007/s43440-025-00806-6

**Published:** 2025-11-19

**Authors:** Madhura M. Bose, Anusha Govindula, Madhavan Nampoothiri, Devinder Arora, Jayesh Mudgal

**Affiliations:** 1 Department of Pharmacology, Manipal College of Pharmaceutical Sciences, Manipal Academy of Higher Education, Manipal, Karnataka 576104 India; 2 School of Pharmacy and Medical Sciences, Gold Coast Campus, Griffith University, Gold Coast, Queensland 4222 Australia

**Keywords:** Treatment-resistant depression, Hypothalamic-pituitary-adrenal axis dysregulation, Blood-brain barrier dysfunction, Kynurenine pathway, Epigenetics, Glucocorticoid receptor resistance

## Abstract

Treatment-resistant depression (TRD) continues to pose a major challenge in clinical practice, as a large proportion of patients fail to achieve remission despite multiple antidepressant drugs. Growing evidence indicates that dysregulation of the hypothalamic-pituitary-adrenal (HPA) axis, together with epigenetic alterations, neuroinflammation, and kynurenine pathway metabolism, plays a central role in the pathophysiology of TRD. Particularly, prolonged stress-induced glucocorticoid receptor (GR) resistance, persistent hypercortisolaemia, and elevated pro-inflammatory cytokines contribute to neurotoxicity, hippocampal atrophy, and impaired neuroplasticity, aggravating depressive symptoms and reducing treatment response. Additionally, dysregulated tryptophan metabolism and the shift towards neurotoxic kynurenine metabolites further impair neuronal function and resulting in TRD. This review integrates recent findings on the complex interplay between HPA axis dysfunction, neuroimmune responses, and metabolic disturbances in TRD while highlighting novel therapeutic avenues such as ketamine, GR modulators, and anti-inflammatory agents. Further, disruption in the blood-brain barrier as one of the mechanisms of TRD was also reviewed. A deeper understanding of these mechanisms will enable the development of personalized treatment strategies to enhance clinical outcomes for TRD patients.

## Clinical trial number

Not applicable.

## Highlights

• HPA axis dysregulation and neuroinflammation point towards TRD.

• Disrupted kynurenine pathway causes neurotoxicity and hippocampal atrophy.

• Resulted metabolites impair treatment responses.

• Conventional therapy fails to address HPA dysfunction and metabolic imbalances.

• Addressing epigenetic and metabolic changes may reverse treatment resistance.

## Introduction

Major depressive disorder (MDD) and the associated mood disorders pose a significant challenge to the psychiatric society. Due to poor diagnosis criteria and social stigma surrounding depression among patients, depressive symptoms have been ingrained in our lifestyle [1]. MDD is a heterogeneous condition that can manifest at any time in life, and approximately 30% of patients are resistant to conventional treatments. Importantly, MDD is frequently associated with non-suicidal self-injury (NSSI) and suicidal behaviors. Bullying (as a victim or offender) further increases the risk of short and long-term adverse outcomes [2]. A comprehensive clinical trial (Sequences Treatment Alternatives to Relieve Depression - STAR*D) has shown that 67% of patients responded after 4 trials of antidepressant treatment. Additionally, 10% to 20% of individuals with MDD continued to experience symptoms even after receiving sequential therapeutic approaches [3, 4].

It is well accepted that the remission rate of commonly used antidepressant drugs is low, with only one-third of patients responding to treatment. These individuals who do not experience significant benefits from the drugs are categorized as having treatment-resistant depression (TRD) [5, 6]. Even with various definitions, there is no clear and comprehensive statement defining TRD, and it is still a significant challenge for the psychiatric community. Many TRD patients are classified as non-responders or partial responders [7]. This situation poses a considerable risk to treatment for TRD patients, including overdosing, leading to various adverse effects. In psychiatry, TRD revolves around the term “resistance,” and hence the definition arises as failure to respond to an adequate dose and duration of antidepressant treatment. However, a standalone therapy with any antidepressant drug fails to remit MDD in patients with co-existing psychiatric disorders [8]. There are significant challenges associated with TRD, including a lack of agreement on its definition, clear criteria for classifying TRD patients, a distinct diagnostic approach, and a potent therapeutic strategy specialized for TRD patients.

TRD affects patients who do not achieve remission after at least two antidepressant trials [9]. When the standard antidepressant drugs or therapeutic strategy fail to achieve the desired outcome, clinicians may opt for considering various other pharmacological/non-pharmacological interventions. Non-pharmacological treatments include psychotherapy (often combined with medications), electroconvulsive therapy (ECT), and vagus nerve stimulation (VNS)—the latter approved by the Food and Drug Administration (FDA) but typically showing benefits only after long-term use. Experimental options, such as repetitive transcranial stimulation, deep brain stimulation, and psychosurgery, are not widely accessible [10–12].

The pharmacological treatment for TRD can be categorized into two main groups: “switching” and “combining”. In the case of the “switching approach,” medications are switched either within or between the existing antidepressant classes. Switching of the drugs offers the advantage of avoiding polypharmacy [13]. However, this approach imposes a drawback of losing the partial benefit gained from the initial treatment when switching to another medication. Additionally, it has been demonstrated that switching to monotherapy offers limited effectiveness in achieving remission [14]. Combination therapies provide a better therapeutic outcome as compared to partial improvement with monotherapy. Several non-antidepressant medications like lithium and thyroid hormones have been extensively studied but are not widely accepted. Switching antidepressants within or between classes can avoid polypharmacy, whereas combination approaches may improve outcomes with a higher risk of adverse effects and financial burden to the patients [15]. In addition, evidence suggests that a combination of atypical antipsychotics and antidepressants offers a better clinical outcome in TRD patients. Emerging psychoactive treatments, including modern pharmacological agents, may help attenuate negative clinical outcomes, including suicidal risk, in patients with TRD [16]. This review aims to explore the complex pathophysiological mechanisms underlying TRD, including dysregulation of the hypothalamic-pituitary-adrenal (HPA) axis, neuroinflammation, epigenetic modifications, and disruptions in the kynurenine pathway. By understanding these biological contributors, the review seeks to shed light on why standard treatments often fail and to highlight emerging therapeutic approaches that hold promise for improving outcomes in patients with TRD.

## Methodology

For this review, a comprehensive literature search was performed in PubMed and Scopus databases using keywords such as ‘HPA axis,’ ‘kynurenine pathway,’ ‘treatment-resistant depression,’ ‘TRD,’ and ‘neuroinflammation.’ Articles published between 2000 and 2025 were considered, including clinical, preclinical, and mechanistic studies. Only peer-reviewed publications in English were included, and studies not directly addressing the neurobiological mechanisms of TRD were excluded. Reference lists of relevant articles were also screened to identify additional pertinent studies.

### Clinical features of treatment-resistant depression (TRD)

Around 300 million people are suffering from MDD. TRD is a subtype of MDD that is depicted by the inadequate response to standard initial treatments [16]. Currently, there is no consensus on various staging models or definitions of TRD, which raises questions regarding the associated mood syndromes. At present, the inefficient diagnostic criteria and the stigma built around depression contribute to the persistence of symptoms. Amidst all this, the crippling symptoms of MDD become a fatal risk to the person experiencing them [17].

TRD faces a major challenge of universal acceptance and clear definition, which eventually highlights a notable concern in translational research, the development of treatment strategies, and the development of healthcare-related policies [18]. Establishing more focused psychiatric treatments necessitates a defined and clear identification of the TRD-related phenotypes. In the absence of a universally accepted definition, clinical trials recruit heterogeneous patient populations, leading to hindrances in interpreting and complicating the applicability to a wider population suffering from the disorder. The variability extends to clinical practice, resulting in differences among patients who do not respond to first-line antidepressants [19]. Such discrepancies in practice can negatively impact therapeutic outcomes, while the absence of a standard definition also affects policy decisions, including reimbursement and treatment accessibility for TRD patients [20]. Regulatory agencies like the FDA and European Medicines Agency (EMA) define TRD as the inability to respond to two or more antidepressant regimens despite following adequate dosage, duration, and adherence [21]. However, due to a lack of clarity and consensus, it is depicted as being associated with the concept of partial response, which ultimately complicates the understanding of the disease and makes it more challenging to comprehend the condition [22]. This profoundly highlights the reality that, despite the acknowledged definitions and understanding of TRD, limitations continue to exist, as there is neither a universally accepted definition nor a well-established criterion for its identification and treatment. Alternative classification models, such as the Thase and Rush staging system, conceptualize TRD along a continuum of treatment failures, ranging from resistance to a single antidepressant trial to resistance even after ECT. Additionally, recent transcriptome-wide association studies (TWAS) have also identified distinct biological traits in individuals with TRD compared to those who respond to treatment [23, 24]. The TWAS findings show that TRD patients have more than twice as many abnormalities in neurotrophic pathways as in monoamine transmission, suggesting that enhancing synaptic plasticity and neurotrophic factors could be a potential treatment for TRD [25].

As there are several definitions of TRD, however, they all share a fundamental characteristic: an inadequate response to at least one adequately dosed and sufficiently prolonged antidepressant trial [26]. Several staging models have been proposed to define the severity and treatment failure levels in TRD (Table 1).

**Table 1:** Staging models for TRD

Model	Definition & criteria	Strengths	Limitations	References
Thase and Rush model	TRD is staged progressively: **Stage I**: Failure of ≥ 1 antidepressant trial. **Stage II**: Failure of ≥ 2 different classes of antidepressants. **Stage III**: Stage II, along with the failure of TCA treatment. **Stage IV**: Stage III, along with the failure of monoamine oxidase inhibitors. **Stage V**: Stage IV, combined with the failure of bilateral ECT.	Defines TRD along a continuum rather than categorically.	Lacks a precise operational definition of “failure.” Does not account for psychotherapy resistance.	[22, 27]
Maudsley staging model	-TRD was assessed using three dimensions: treatment failure, episode duration, and severity. -Treatment failure (0–7 points): Failure of multiple medications, augmentation, or ECT. -Episode duration (0–3 points): Acute ( < 12 months), subacute (13–24 months), or chronic ( > 24 months). -Severity (0–5 points): Ranges from subsyndromal to severe with psychosis. Total score classification: mild (3–6), moderate (7–10), severe (11–15).	Includes illness duration and severity, aiding outcome prediction.	Arbitrary scoring and a lack of validation for the weighting system. Does not define “failure” explicitly.	[27]
Dutch measure for TRD	-Expands MSM by adding functional impairment, anxiety, personality disorders, psychosocial stressors, and treatment intensity. -Scoring: Functional impairment (0–3), comorbid anxiety (0–1), augmentation therapy (0–3), psychotherapy use (0–2), inpatient treatment (0–2). -Max Score: 27.	Most comprehensive; it considers multiple psychosocial and treatment variables.	Does not include physical comorbidities or childhood adversities. Low threshold for TRD diagnosis.	[22, 27]
Massachusetts general hospital staging model	-One point per failed adequate antidepressant trial. -Half point per optimization of dose/duration or augmentation. -Three points for failure of ECT.	Accounts for treatment optimization. No hierarchy of antidepressant classes.	Arbitrary scoring: equal weight for dose optimization and augmentation strategies is not empirically validated.	[27]
European group for the study of resistant depression	-Defines TRD as failure to respond to two or more adequate trials of different antidepressant classes. -Non-response: < 50% reduction in HAM-D or MADRS score. -Subcategories: Includes chronic resistant depression (≥12 months).	Clear definition of non-response based on HAM-D/MADRS. No assumed hierarchy of antidepressants.	Chronic depression is defined as ≥ 12 months, which is shorter than the typical 2-year standard.	[27]

TRD, treatment-resistant depression; TCA, tricyclic antidepressant; ECT, electroconvulsive therapy; MSM, maudsley staging model; HAM-D, hamilton depression rating scale; MADRS, montgomery–åsberg depression rating scale

TRD is centered around the concept of “resistance,” thus leading to a definition as the failure to respond to antidepressant treatment given at an appropriate dosage and for a sufficient duration [27, 28]. The absence of a standardized definition for TRD hinders the progress of mechanistic and translational research, delaying the identification of innovative and personalized treatment options. It is important to point out that the prevalence of TRD in real-world practice is imprecise but is expected to increase because of gaps in knowledge application, challenges in accessing necessary healthcare, and the intricacy of how the disease manifests. Over half of those suffering from depression do not react positively to standard first-line treatments, and around 30% do not respond to multiple attempts with various antidepressant drugs [27, 29]. Various conventional and emerging treatment approaches are currently employed to manage TRD, ranging from pharmacotherapy to neuromodulation techniques (Table 2). Significant adverse effects from some TRD treatments may restrict their usage or force the termination of otherwise potentially beneficial medications. Personalized techniques are essential since patients respond to treatment differently from one another. In general, improving diagnostic standards and treatment approaches can be facilitated by deepening our understanding of TRD [30].

**Table 2:** Current therapeutic strategies for TRD

Therapeutic strategy	Description	Key findings	References
Optimization strategies	Insufficient treatment trials lead to “pseudo-resistance.” Treatment response should be reassessed every 3–4 weeks, and dosage should be optimized before concluding treatment resistance.	Helps differentiate pseudoresistant from true TRD by maximizing the dose and duration of treatment.	[27, 28]
Augmentation	Adding a second drug (non-antidepressant) to a primary antidepressant therapy.	Risk of misdiagnosing inadequate medication response as TRD.	[27, 29]
Switching strategies	Shifting from one antidepressant class (e.g., SSRI/SNRI) to another when first-line options fail.	70% of patients who couldn’t tolerate one SSRI responded to a second SSRI.	[29, 30]
Combinatorial approach	Combining SSRIs with heterocyclic antidepressants or tetracyclic antidepressants after initial failure.	55% of patients responded positively to combination therapy in a small-scale trial.	[30]
Lithium	Lithium augmentation is historically used in mental health treatment.	Lithium increases favorable outcomes from 30% to 70%, with an 88.5% reduction in suicide risk.	[27, 31]
Psychotherapy	Various psychotherapeutic interventions, such as cognitive behavioral therapy, interpersonal therapy, and problem-solving therapy.	Effective for patients with specific symptoms and stressors; preferred by many patients over medication. Mixed results, but psychotherapy helps alter patients’ perception of their illness.	[31]
Electroconvulsive therapy (ECT)	Used when multiple treatment attempts fail. Alters serotonin receptor expression and brain wave activity. 50–60% response rate, quick improvement for	Maintenance ECT or medication needed post-ECT	[27, 29]
Vagus nerve stimulation (VNS)	Electrical pulses are delivered to the vagus nerve via an implanted generator. 33–35% of TRD patients showed symptom relief.	Long onset time; may obstruct the recall of negative information	[27, 30]
Repetitive transcranial magnetic stimulation (rTMS)	Magnetic pulses stimulate the brain to alleviate depression. Response rates between 30.6% and 64.7%.	Requires 4–6 weeks for significant results	[27, 31]
Deep brain stimulation	Neurosurgical implant stimulating specific brain regions. 4 out of 6 patients in a study achieved remission.	High risk; should be a last resort	[30, 31]
Theta-burst stimulation (TBS)	A form of rTMS that mimics natural brain theta rhythms for cortical plasticity. Uses short bursts of high-frequency stimuli.	Suggested as a promising TRD treatment.	[30]

TRD, treatment-resistant depression; SSRI, selective serotonin reuptake inhibitor; SNRI, serotonin–norepinephrine reuptake inhibitor; ECT, electroconvulsive therapy; VNS, vagus nerve stimulation; rTMS, repetitive transcranial magnetic stimulation; TBS, theta-burst stimulation

### Neurobiology of TRD: understanding resistance to antidepressant therapy

The precise mechanism underlying TRD is unclear. Emerging research suggests that multiple biological, genetic, and psychosocial factors contribute to its development. Therefore, understanding these factors is crucial in developing targeted interventions and improving treatment outcomes against TRD [31–33]. Specifically, the etiopathology of TRD involves disruptions in neurotransmitter systems, HPA axis dysfunction, neuroinflammation, and impaired neuroplasticity, including deficits in synaptic function and connectivity [34]. Genetic predisposition, epigenetic modifications, and environmental stressors such as chronic stress/early-life trauma may further heighten TRD. Moreover, individual variability in drug metabolism, pharmacokinetic properties, and the presence of psychiatric or medical comorbidities can impact treatment response. A deeper understanding and knowledge of these mechanisms is highly essential for developing targeted treatment strategies, identifying predictive biomarkers, and optimizing interventions to improve clinical outcomes in TRD patients all over the world [35].

#### Axis dysregulation in TRD

A range of internal and external stressful factors can disrupt the body’s homeostasis, triggering an “adaptive stress response” to reestablish balance. The HPA axis plays a crucial role in the body’s stress response, regulating energy balance, immune function, and cognitive processes [30, 36]. Cortisol, a steroid hormone released from adrenal gland in response to HPA activation, helps to manage stress by mobilizing energy reserves, modulating immune responses, to influencing mood and cognition. Once the stressor subsides, cortisol provides negative feedback to the hypothalamus and pituitary to restore balance [10].

In addition to HPA axis activation, stress also engages the autonomic nervous system, particularly the vagus nerve. Through the cholinergic anti-inflammatory pathway (CAIP), the vagus nerve modulates immune responses and inflammatory processes. This neural-immune interaction represents a critical mechanism linking stress resilience, inflammatory regulation, and the pathophysiology of TRD. [37–39]. Stressors trigger hypothalamus to release the corticotropin-releasing hormone (CRH). This activates the posterior pituitary gland to release the adrenocorticotropic hormone (ACTH) and stimulating the noradrenergic neurons of the locus coeruleus/norepinephrine (LC/NE) system. The LC/NE system plays a crucial role in initiating a “fight or flight” response, driven by epinephrine and norepinephrine, while ACTH upregulates the production of cortisol from the adrenal cortex [40].

Chronic stress can cause disruption of the normal functioning of the HPA axis, leading to altered cortisol secretion and disrupted organ function. Dysregulation of this system due to various stressors and psychiatric disorders is associated with significant changes in the brain’s stress-regulatory mechanisms, contributing to mood and anxiety disorders [41]. Early life stress disrupts the normal function of the HPA axis, leading to abnormal cortisol secretion and impairing the system’s ability to regulate stress. Prolonged cortisol imbalance can shrink hippocampal volume, reduce neurogenesis, and weaken the brain’s stress response, increasing vulnerability to mood and anxiety disorders. Exposure to life stressors is one of the most pertinent precipitating factors in developing depressive episodes [42–44].

Although alterations in the HPA axis may vary according to the subtypes of MDD, scientific literature has shown that persistent stress disrupts the axis feedback system due to excessive secretion of CRH and ACTH, leading to impaired negative feedback mechanisms by glucocorticoids [45–47]. As a consequence, there is sustained cortisol elevation and insufficient suppression of HPA axis activity owing to dysregulated glucocorticoid receptor (GR) sensitivity, that interferes with the negative feedback mechanism. Moreover, prolonged exposure to elevated cortisol levels initiates glucocorticoid resistance by downregulating GR expression and signaling. Additionally, pro-inflammatory cytokines namely IL-6 and tumor necrosis factor (TNF)-α further interfere with GR signaling, making HPA axis dysfunction worse and impairing the body’s capacity to regulate cortisol levels effectively. This cascade ultimately aids in sustained hypercortisolism and diminished stress resilience in TRD [47, 48]. Individuals clinically diagnosed with MDD exhibited higher scores for both depression and stress-related symptoms. They also had significantly elevated cortisol levels in comparison to the control subjects. Furthermore, depression exhibited a positive correlation with stress scores [49]. These findings suggest that chronic stress is involved in elevated cortisol levels, which, at least in part, appears to be the underlying mechanism of MDD in these individuals. Until now, an established and suitable mechanism has not been developed that can be portrayed as the reason for HPA axis dysregulation and explain the aspects of TRD [50]. However, it was observed that the development of MDD is the result of environmental and genetic interactions. Various factors are the reason for the development of MDD, and few of them are attributed to early life stress, such as sexual, emotional, mental, or physical trauma. Adverse events like loss of loved ones, unemployment, financial constraints, educational stress, childhood sexual abuse, family disputes, separation, and life-threatening health problems often arise in the year preceding the MDD onset [51]. The HPA axis dysregulation is promptly shaped by childhood trauma and unforgettable experiences. These traumas lead to heightened sensitivity thus leading to an increased response to subsequent stressors. The HPA axis and the kynurenine pathway (KP) are intricately connected in TRD. Stress activates the HPA axis, leading to elevated glucocorticoid levels, which influence kynurenine metabolism by upregulating enzymes such as indoleamine 2,3-dioxygenase (IDO) [52]. Subsequently, increased quinolinic acid (QUIN) levels result in neurotoxicity through N-methyl-D-aspartate (NMDA) stimulation. In contrast, inflammatory cytokines such as IL-6 also activate the kynurenine pathway by overexpressing IDO and thereby leading to overproduction of QUIN, which is a neurotoxic metabolite. This metabolite, in turn, can affect HPA axis function by altering GR sensitivity, creating a feedback loop that exacerbates depressive symptoms. This bidirectional interaction emphasizes the complex interplay between stress, inflammation, and metabolic dysregulation in the pathophysiology of depression [53, 54]. Beyond stressful life events and trauma, genetic factors also play a role. For example, variants of FKBP5, a co-chaperone of heat shock protein 90 (HSP90) and an important regulator of HPA axis activity, have been shown to affect GR sensitivity [55]. The FKBP5 genetic variants affect the GR sensitivity, thereby contributing to HPA axis dysregulation. These genetic variants are seen to reduce the GR sensitivity causing impairment in the negative feedback inhibition of the HPA axis leading to prolonged cortisol release and hyperactivation of the HPA axis, which is frequently seen in stress-associated conditions such as TRD [55, 56]. The contributing factors like ELS and stressful events later in life may lead to treatment resistance in MDD patients by possibly impairing the HPA axis function [57]. An important phenomenon in TRD is the so-called “glucocorticoid paradox”. While glucocorticoids typically function as anti-inflammatory hormones via GR activation, patients with depression often exhibit elevated cortisol levels alongside persistent inflammation. This apparent contradiction can be explained by GR resistance, whereby receptor function is impaired by chronic stress and pro-inflammatory signaling. As a result, the negative feedback regulation of the HPA axis is blunted, leading to sustained hypercortisolemia and immune activation. This paradox highlights the dysfunctional interplay between stress hormones and inflammatory pathways in the pathophysiology of TRD [58].

In depression, elevated cortisol levels and impaired GR function have been associated with excessive stress response and structural alterations by attenuating the HPA axis-induced negative feedback inhibition in key brain areas like the hippocampus, amygdala, and prefrontal cortex that are associated with mood dysregulation and cognitive impairment in depression [59]. As a consequence, elevated cortisol level leads to neurodegeneration and neuroinflammation. Notably, HPA axis dysregulation is widely observed in TRD patients, where the conventional treatment fails to respond and restore the stress hormone regulation. Studies have depicted that non-responders to antidepressants often show persistent GR resistance and cortisol hypersecretion, suggesting that the failure to normalize the normal functioning of the HPA axis will lead to the development of TRD symptoms [60, 61].

Studies comparing HPA axis function in treatment-resistant unipolar depression (TRUD) and treatment-resistant bipolar depression (TRBD) suggest a unique form of dysregulation, which contributes as the primary reason for antidepressant resistance. Patients with TRUD have demonstrated HPA axis hyperactivity, which is usually seen by increased cortisol levels, indicating a stagnant stress response and incapability in regulating the glucocorticoid feedback [62]. In contrast to the TRBD patients who exhibit HPA axis hypoactivity with a blunted cortisol awakening response (CAR), representing an unprompted biological response to chronic stress. The presence of HPA axis abnormalities in TRD demonstrates that cortisol-modulating therapies such as GR antagonists (e.g., mifepristone), corticotropin-releasing factor antagonists (e.g., pexacerfont), and cortisol synthesis inhibitors (e.g., ketoconazole) may illustrate immense therapeutic potential in restoring neuroendocrine balance [63]. Sustained activation of the HPA axis and chronic stress can lead to excessive cortisol production, which in turn can lead to factors like neurotoxicity, impaired neuroplasticity, and hippocampal atrophy, showing the symptoms of TRD. These patients commonly exhibit elevated cortisol levels, indicating that maladaptive stress response hinders the efficacy of the treatment strategy [64]. Hypercortisolism plays a role in antidepressant resistance by weakening GR function, compromising neuroplasticity, facilitating neuroinflammation, and altering monoamine neurotransmission, eventually diminishing the therapeutic impact of the standard treatment approaches. The abnormalities suggest that dysregulation of the body’s stress response system contributes significantly to depressive symptoms [65]. The various mechanisms and consequences of HPA axis dysregulation in patients with TRD are summarised (Table 3).

**Table 3:** HPA axis dysregulation in patients with TRD

Aspect	Findings in TRD	Implications	References
Cortisol levels	Elevated basal cortisol, blunted diurnal rhythm	Increased stress response, impaired emotional regulation	[59]
GR resistance	Reduced GR sensitivity to cortisol feedback	Sustained HPA axis hyperactivity, decreased antidepressant efficacy	[58, 59]
CRH and ACTH levels	Increased ACTH and CRH	Overactivation of the HPA axis, heightened stress reactivity	[59]
Hippocampal atrophy	Reduced hippocampal volume due to prolonged cortisol exposure	Impaired mood regulation, cognitive dysfunction, poor treatment response	[58, 59]
Inflammatory cytokines	Increased IL-6, TNF-α, and IL-1β	Neuroinflammation, dysregulation of HPA axis and neurotransmission	[60, 61]
Dex/CRH test response	Blunted suppression of cortisol following dexamethasone challenge	Defective negative feedback, persistent HPA axis overactivity	[62]
HPA-targeted therapeutic strategies	GR modulators (mifepristone), CRH antagonists (verucerfont), anti-inflammatory agents (minocycline)	Potential interventions to restore HPA function and improve treatment outcomes	[59, 63, 64]

where TRD, treatment-resistant depression; HPA, hypothalamic–pituitary–adrenal; GR, glucocorticoid receptor; CRH, corticotropin-releasing hormone; ACTH, adrenocorticotropic hormone; IL, interleukin; TNF-α, tumor necrosis factor-alpha; Dex/CRH, dexamethasone/corticotropin-releasing hormone

#### Kynurenine pathway dysregulation in TRD

**Fig. 1:**
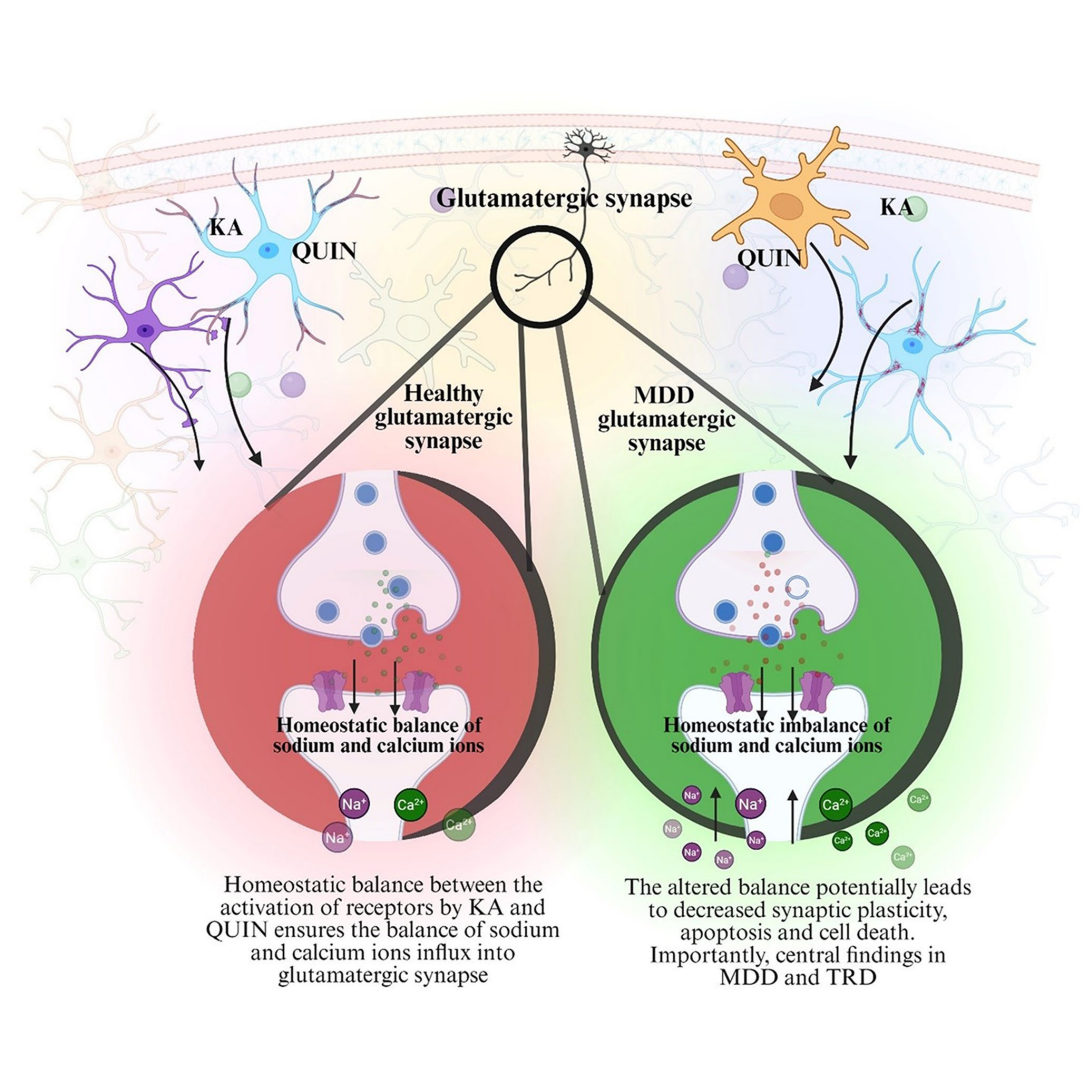
Homeostatic imbalance of KA and QUIN leading to TRD. The imbalance between neuroprotective KA and neurotoxic QUIN of the kynurenine pathway. In TRD, excessive QUIN production promotes excitotoxicity and neuroinflammation, while reduced KA weakens neuroprotection. The resulting homeostatic disruption contributes to impaired neuronal function, persistent depressive symptoms, and poor treatment response. KA, kynurenic acid; MDD, major depressive disorder; QUIN, quinolinic acid; TRD, treatment-resistant depression

The kynurenine pathway (KP) is known to play a crucial role in the development of MDD and has also been associated with TRD. This pathway is significantly linked to neuropsychiatric and neurodegenerative conditions, where an imbalance between its neurotoxic and neuroprotective metabolites has been reported in individuals with MDD (Fig. 1). Specifically, studies have found increased plasma kynurenine levels alongside decreased kynurenic acid (KA) concentrations in these patients [66]. Activation of the KP, often triggered by chronic stress or inflammatory processes, leads to the production of various neuroactive metabolites, including the neurotoxic compound, quinolinic acid (QUIN). Elevated levels of these harmful metabolites in TRD patients suggest that KP dysregulation may contribute to resistance against conventional antidepressant treatments [67]. As a fundamental biochemical pathway for tryptophan metabolism, KP results in the synthesis of key neuroactive compounds such as KA and QUIN. Research has indicated that an imbalance within this pathway is strongly associated with TRD [68]. Chronic stress and persistent inflammation may divert tryptophan metabolism toward increased QUIN production, resulting in higher levels of this neurotoxic metabolite. Since QUIN acts as an NMDA receptor agonist, it exacerbates neuroinflammation and contributes to neuronal damage [69]. At the same time, there is a notable reduction in KA, a neuroprotective and anti-inflammatory molecule. This metabolic shift is believed to play a role in the persistence of depressive symptoms and the diminished effectiveness of traditional antidepressant treatments [70].

Beyond the primary kynurenine metabolites, several key enzymatic pathways shape the balance between neurotoxicity and neuroprotection. Kynurenine monooxygenase (KMO), predominantly expressed in microglia, converts kynurenine into 3-hydroxykynurenine and QUIN, both associated with excitotoxicity and oxidative stress. In contrast, kynurenine aminotransferases (KATs), largely located in astrocytes that facilitate the formation of KA, which acts as an NMDA receptor antagonist with neuroprotective properties [71]. Thus, the relative activity of KMO versus KATs determines whether the kynurenine pathway shifts toward neurotoxic or neuroprotective outcomes. Dysregulation of this enzymatic balance, with increased microglial KMO activity, may favor the accumulation of neurotoxic metabolites and contribute to treatment resistance in depression [72, 73].

Chronic inflammation can activate the primary glial cells present in the central nervous system, that is, microglia and astrocytes, which thereby results in the release of pro-inflammatory cytokines. This response disrupts glutamate metabolism, causing excess extracellular glutamate and excitotoxicity, both of which contribute to TRD. This further highlights the interconnection between inflammation, glutamate neurotransmission, and glial cell function in the onset and continuation of mood disorders, including TRD [74]. A key player in this process is IDO-1, an enzyme that leads the conversion of tryptophan into kynurenine. Pro-inflammatory cytokines, particularly interleukin (IL)-6, strongly upregulate IDO-1 activity through the JAK-STAT signaling pathway, increasing its expression. This induction results in increased tryptophan degradation and elevated kynurenine production, processes that have been implicated in the pathophysiology of TRD. Furthermore, genetic polymorphisms in the IDO-1 gene may modulate its expression and activity, potentially influencing individual susceptibility to TRD and response to treatment [75, 76]. The KP has a significant impact on TRD. Chronic inflammation in the body can activate the pathway and change the mechanism of tryptophan which is toxic to brain cells as it hyperactivates NMDA receptors, giving rise to brain inflammation and damage. Instead of producing helpful and protective substances like KA, the pathway initiates the formation of harmful metabolites like QUIN. KA protects the brain by blocking these receptors and reducing inflammation. However, in TRD, inflammation causes the body to make less KA and more QUIN, increasing toxicity and making depression harder to treat [77].

**Fig. 2:**
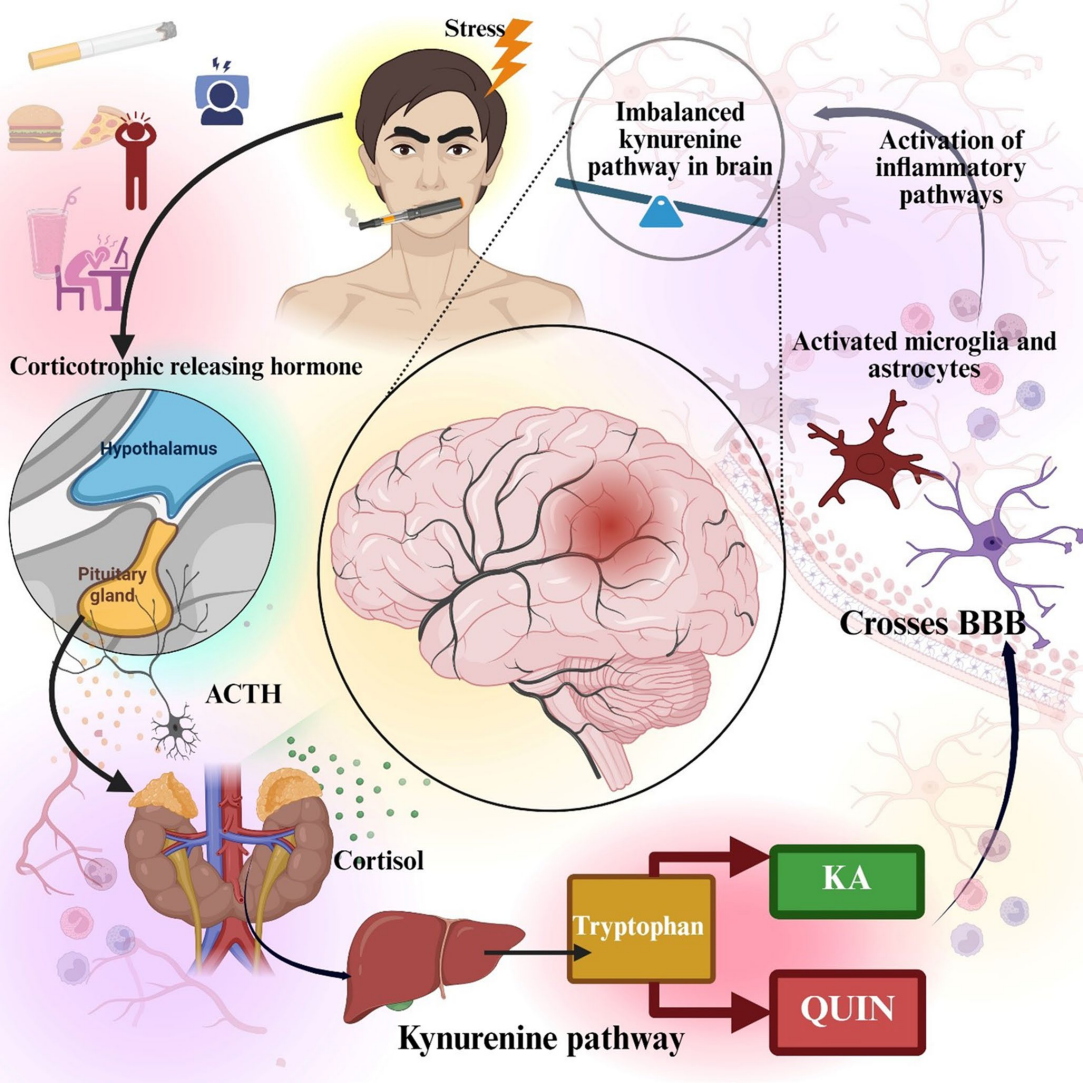
Impact of the HPA axis, kynurenine pathway, and blood–brain barrier in TRD. Prolonged stress leads to excessive cortisol production, disrupting peripheral kynurenine metabolism to generate excess of neurotoxic metabolite, quin. Imbalanced kynurenine pathway in brain due to activated microglia and subsequent neuroinflammation leads to TRD. Together, these processes create a cycle of impaired neuroplasticity, persistent inflammation, and inadequate antidepressant response. HPA, hypothalamus-pituitary-adrenal; KA, kynurenic acid; QUIN, quinolinic acid; TRD, treatment-resistant depression

Studies have evidently depicted the interconnection and link of the dysregulation of the tryptophan metabolism with neuropsychiatric disorders, including TRD [78]. Aminocarboxymuconate-semialdehyde decarboxylase (ACMSD) is a potent enzyme in the kynurenine pathway (KP) that is having the function to regulates tryptophan metabolism. Its leading function is to stimulate metabolic intermediates away from developing the neurotoxic compound like QUIN and toward generating the neuroprotective molecule picolinic acid. Dysfunction in ACMSD may interfere with this balance, leading to an overproduction of neurotoxic metabolites while reducing neuroprotective ones, thus worsening depressive symptoms and diminishing the effectiveness of standard antidepressant therapies. Findings further suggest that individuals experiencing heightened inflammation may exhibit alterations in kynurenine metabolism, potentially playing a role in TRD development [45, 77, 79]. The dysregulation of the HPA axis, alterations in the KP, and disruption of the BBB interact to drive the pathophysiology of TRD (Fig. 2).

#### Blood-brain barrier dysfunction in TRD: targeting p-glycoprotein for enhanced antidepressant efficacy

The blood-brain barrier (BBB) is a highly selective protective barrier which helps in the passage of various essential substances in the central nervous system (CNS), ensuring to provide a stable and safe environment for neuronal function. The BBB is formed by tight junction proteins, endothelial cells, pericytes, and astrocytes, which together make sure to provide a safe exchange of molecules between blood and the brain. Prolonged stress and persistent inflammatory actions can disrupt the mechanism and lead to the inefficiency of drug transporters, causing reduced drug efficacy of the antidepressant therapies in TRD patients [80]. The development of antidepressant resistance is related to blood–brain barrier BBB dysfunction due to its restriction of drug permeability, leading to neuroinflammation, and interfering with neurotransmitter balance [81]. The primary mechanisms by which BBB dysfunction interferes with drug delivery and contributes to TRD resistance in patients (Table 4). A contributing factor is the overexpression of efflux transporters, particularly P-glycoprotein (P-gp), which is a multidrug transporter at the BBB that restricts the entry of several antidepressants, including selective serotonin reuptake inhibitors (SSRIs) and tricyclic antidepressants [91]. This limited penetration can lead to insufficient drug concentrations in the brain and, finally cause treatment failure. Clinical and genetic studies have shown that patients with elevated P-gp activity or polymorphisms in the ABCB1 gene (which encodes P-gp) often exhibit weaker responses to antidepressant therapy. Experimental evidence further supports this mechanism: acute inhibition of P-gp with agents such as verapamil enhances the behavioral effects of escitalopram in animal models [92]. Similarly, chronic P-gp inhibition has been reported to increase escitalopram brain levels, suggesting that targeting P-gp could represent a promising augmentation strategy for managing antidepressant-resistant depression [93]. BBB integrity is often seen to be compromised in TRD patients. Persistent stress, elevated glucocorticoids, and inflammatory cytokines such as IL-6 and TNF-α can stimulate BBB permeability, leading to the peripheral immune signals to cross the CNS and contribute to neuroinflammation. Several studies previously cited in Table 4 support these mechanisms, demonstrating that BBB dysfunction may play a key role in the persistence of depressive symptoms and reduced responsiveness to antidepressant therapy [94].

**Table 4:** Blood–brain barrier dysfunction in patients with TRD

Aspect	Mechanism	Impact on TRD	Potential therapeutic strategies	References
Efflux transporter overexpression	Increased activity of P-gp and other efflux pumps (e.g., BCRP, MRP1) actively removes antidepressants from the brain.	Reduced drug accumulation in the CNS, leads to poor therapeutic response.	-P-gp inhibitors (e.g., verapamil, cyclosporine, tariquidar) -Use of P-gp non-substrate antidepressants (e.g., vortioxetine, agomelatine)	[82]
Neuroinflammation and BBB disruption	Elevated levels of pro-inflammatory cytokines IL-1β, TNF-α, interferon γ disrupt tight junction proteins (e.g., claudin-5, occludin, ZO-1).	Increased BBB permeability allows neurotoxic substances to enter the brain, worsening depressive symptoms.	-Anti-inflammatory agents (e.g., COX-2 inhibitors, minocycline, statins) -Omega-3 fatty acids, curcumin, and flavonoids to restore BBB integrity	[83, 84]
Kynurenine pathway activation	Chronic inflammation increases IDO activity, leading to accumulation of neurotoxic metabolites (QUIN, 3-hydroxykynurenine).	Neurotoxic effects contribute to neuronal dysfunction and antidepressant resistance.	- IDO inhibitors (e.g., 1-methyl-D-tryptophan) - Kynurenine pathway modulators (e.g., ketamine, N-acetylcysteine)	[45, 85, 86]
HPA axis dysregulation	Chronic stress leads to excessive cortisol secretion, which disrupts tight junctions and increases BBB permeability.	Cortisol-induced BBB dysfunction allows peripheral inflammatory signals to enter the brain, exacerbating TRD.	- GR antagonists (e.g., mifepristone) - CRH receptor antagonists to stabilize BBB function	[87, 88]
Epigenetic modifications	DNA methylation and histone acetylation alter tight junction-related genes and efflux transporter expression.	Increases BBB permeability and enhances drug efflux, leading to reduced antidepressant effectiveness.	-Epigenetic drugs (e.g., HDAC inhibitors) to restore BBB function -Lifestyle interventions (e.g., exercise, diet) that influence epigenetic changes	[89]
Nanotechnology-based drug delivery	Conventional antidepressants struggle to penetrate the dysfunctional BBB.	Limited CNS bioavailability of antidepressants worsens treatment outcomes.	-Lipid-based nanoparticles (liposomes, micelles, exosomes) -Intranasal and polymeric nanoparticle formulations to bypass the BBB	[90]

BBB, blood–brain barrier; P-gp, p-glycoprotein; BCRP, breast cancer resistance protein; MRP1, multidrug resistance-associated protein 1; CNS, central nervous system; IL, interleukin; TNF-α, tumor necrosis factor-alpha; ZO-1, zonula occludens-1; COX-2, cyclooxygenase-2; IDO, indoleamine 2,3-dioxygenase; QUIN, quinolinic acid; HPA, hypothalamic–pituitary–adrenal; GR, glucocorticoid receptor; CRH, corticotropin-releasing hormone; HDAC, histone deacetylase

Impairment of BBB is often seen due to overexpression of the neuroinflammatory markers, such as pro-inflammatory cytokines, which leads to disruption of the tight junction proteins and causes increased permeability. High inflammatory metabolite thus activates indolamine 2,3-dioxygenase (IDO) enzyme, which eventually leads to upregulation of KP metabolites such as QUIN, resulting in neurotoxicity and TRD [95]. It is equally important to understand that high cortisol production due to the result of prolonged chronic stress can lead to disruption of the tight junction integrity and enhance BBB permeability, which enables the penetration of inflammatory cytokines into the brain more easily. Cortisol also modulates GR signaling, which can influence the expression of P-gp and other drug transporters, leading to a reduction in the effectiveness of antidepressants [96]. In conclusion, BBB dysfunction plays a primary role in TRD by impairing drug penetration, enhancing neuroinflammation, and disrupting neurotransmitter balance. Future research focusing on efflux transporter modulation, anti-inflammatory treatments, and advanced drug delivery systems could lead to more effective therapeutic strategies for patients with TRD [97, 98]. Disruption of the BBB is increasingly recognized as a key contributor to the pathophysiology of TRD. BBB dysfunction allows peripheral inflammatory cytokines, to enter the central nervous system, triggering neuroinflammation and altering neuronal signaling. Persistent stress and impaired HPA axis activity can further enhance this permeability, developing a feedback loop that sustains depressive symptoms and reduces responsiveness to conventional antidepressant treatments. This mechanism highlights the critical role of neurovascular integrity in maintaining brain homeostasis and influencing treatment outcomes in TRD [99].

**Fig. 3:**
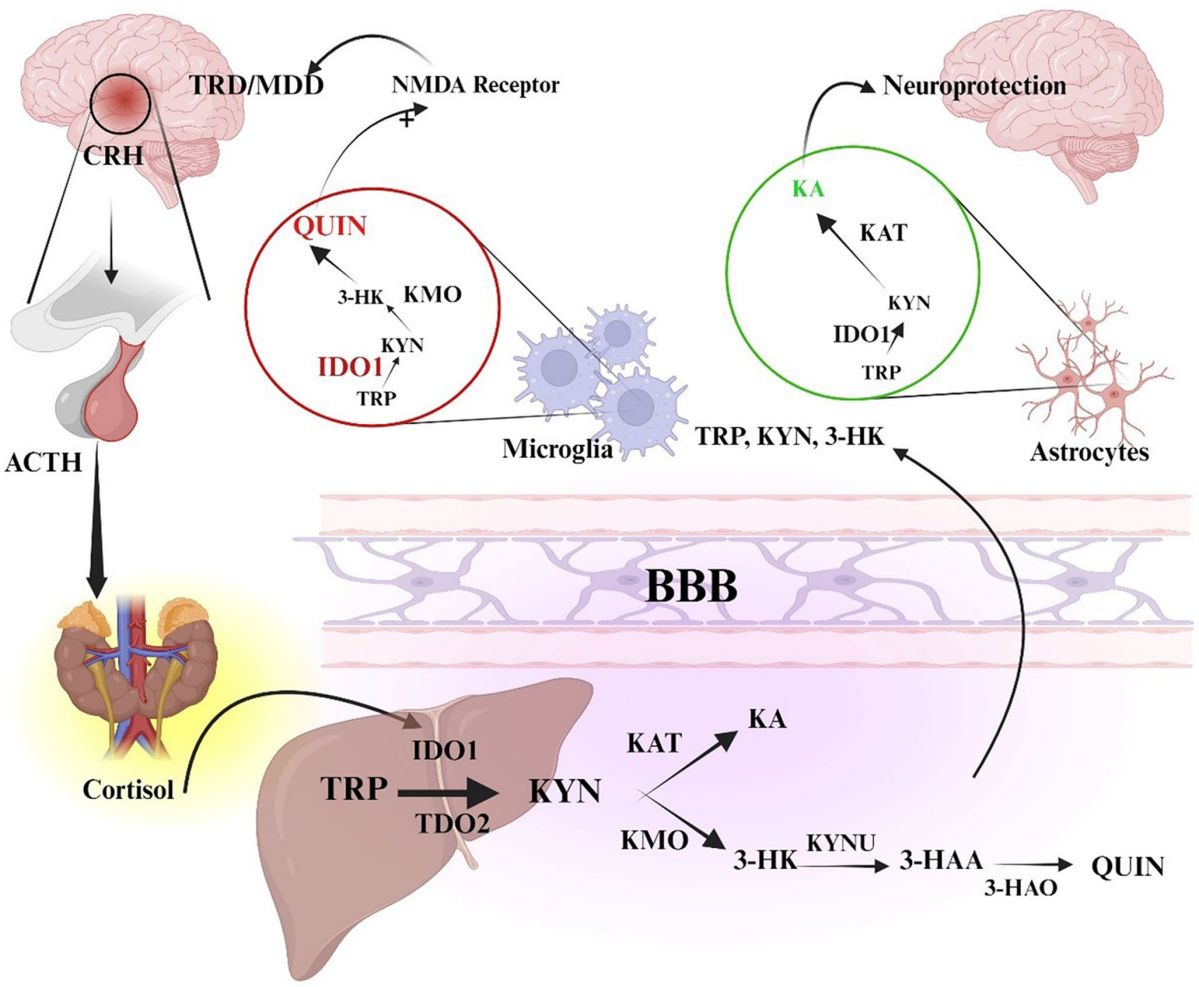
Role of the kynurenine pathway in stress, neuroinflammation, and TRD. ACTH, adrenocorticotropic hormone; BBB, blood-brain barrier; CRH, corticotropin-releasing hormone; IDO1, indoleamine 2,3-dioxygenase 1; KA, kynurenic acid; KAT, kynurenine aminotransferase; KMO, kynurenine monooxygenase; KYN, kynurenine; KYNU, kynureninase; MDD, major depressive disorder; NMDA-N-methyl-D-aspartate; TDO2, tryptophan 2,3-dioxygenase; TRD, treatment-resistant depression; TRP, tryptophan; QUIN, quinolinic acid; 3-HAA, 3-hydroxyanthranilic acid; 3-HK, 3-hydroxykynurenine; 3-HAO, 3-hydroxyanthranilate 3,4-dioxygenase

The kynurenine pathway is increasingly recognized as an important bridge between chronic stress, immune activation, and treatment-resistant depression (TRD) (Fig. 3). When the body is under chronic stress, the hypothalamus releases CRH, which stimulates the pituitary gland to secrete ACTH. This further leads to triggering of the adrenal glands to release cortisol. High levels of this cortisol, in addition to inflammatory signals, activate the enzymes IDO-1 and tryptophan 2,3-dioxygenase (TDO2). These enzymes help to shift the metabolism of tryptophan away from serotonin production and toward kynurenine (KYN) formation, leading to the kynurenine pathway [100]. In microglial cells, kynurenine is metabolized by KMO into 3-hydroxykynurenine (3-HK), which is further processed by kynureninase and 3-hydroxyanthranilate oxidase (3-HAO) to generate QUIN. QUIN is neurotoxic, as it overstimulates NMDA receptors and contributes to excitotoxic damage linked to MDD and TRD [101]. On the other hand, in astrocytes, kynurenine is converted by KATs into KA, which blocks NMDA receptors and offers neuroprotective effects. An imbalance in this pathway, where production shifts toward QUIN instead of KA, appears to worsen depressive symptoms and reduce responsiveness to standard antidepressant treatments. Because of this, targeting specific enzymes in the kynurenine pathway is now being explored as a potential therapeutic strategy for TRD [102].

### Novel therapeutic approaches for TRD

Novel therapeutic approaches for TRD have received increasing attention in recent years. The novel and emerging therapies for TRD are summarized (Table 5), which highlights their mechanisms of action, key findings, and current status. Ketamine, an NMDA receptor antagonist, upregulates glutamate transmission and synaptic plasticity producing rapid antidepressant effects within hours of intravenous infusion with lasting effects for a longer period. Additionally, it also significantly reduces suicidal ideation and is now FDA -approved in the form of intranasal esketamine used alongside oral antidepressants [103, 104]. Psilocybin, a serotonin (5-HT2A) receptor agonist, enhances neuroplasticity and disrupts maladaptive thought patterns, improving depressive symptoms—particularly when combined with psychotherapy—while reorganizing brain connectivity and offering potential long-lasting effects; it is currently under clinical trials with promising outcomes in TRD [105, 106]. Intermittent theta burst stimulation (iTBS), a non-invasive neuromodulatory technique that modulates neural activity and plasticity, has also recently received FDA approval for TRD, showing sustained antidepressant effects with repeated sessions in patients unresponsive to medications [107]. Another emerging direction focuses on mitochondrial bioenergetics, as dysfunction in energy metabolism and oxidative stress contributes to TRD pathophysiology; targeting mitochondrial function through metabolic modulators, antioxidants, or lifestyle-based interventions may pave the way for personalized treatment strategies, although current clinical applications remain limited and require further validation [108, 109].

**Table 5:** Novel therapeutic approaches for TRD

Therapy	Mechanism of action	Key findings	Current status	References
Ketamine	NMDA receptor antagonist; enhances glutamate transmission and synaptic plasticity.	- Rapid antidepressant effects within hours of IV infusion. - Reduces suicidal thoughts significantly in TRD patients. - Effects last for several days post-infusion.	- FDA -approved intranasal Esketamine for TRD. - Used in combination with oral antidepressants.	[103, 104]
Psilocybin	5-HT2A receptor agonist; enhances neuroplasticity and disrupts negative thought patterns.	- Improves depression symptoms, often in combination with psychotherapy. - Helps in the reorganization of brain connectivity. - Potential for long-lasting antidepressant effects.	- Currently under clinical trials. - Shows promising results in TRD treatment.	[105, 106]
Intermittent theta burst stimulation (iTBS)	Non-invasive brain stimulation that modulates neural activity and plasticity.	- Recently FDA-approved for TRD. - Can produce sustained antidepressant effects with repeated sessions.	- Used as a neuromodulatory approach for patients unresponsive to medications.	[107]
Mitochondrial bioenergetics for personalized treatment strategies in TRD	Dysfunction in mitochondrial energy metabolism leads to impaired ATP production and oxidative stress, contributing to neurobiological alterations in TRD.	Targeting mitochondrial function may help develop personalized treatment strategies, improving energy homeostasis and reducing depressive symptoms.	Ongoing research is investigating mitochondrial-targeted interventions such as metabolic modulators, antioxidants, and lifestyle-based approaches. However, clinical applications remain limited, and more studies are needed to validate efficacy in TRD.	[108, 109]

NMDA, N-methyl-D-aspartate; IV, intravenous; FDA, Food and drug administration; TRD, treatment-resistant depression; 5-HT2A, 5-hydroxytryptamine 2A receptor; iTBS, intermittent theta burst stimulation; ATP, adenosine triphosphate

## Conclusion and future perspectives

TRD arises from the combined effects of neurobiological, genetic, and environmental factors. Disrupted HPA axis dysfunction, persistent inflammation, and abnormal kynurenine pathway activity contribute to long-term or sustained depressive symptoms and reduced responsiveness to conventional antidepressant drugs. A deeper understanding of these underlying mechanisms can help the development of personalized treatment strategies and guide the development of emerging therapeutic interventions, including anti-inflammatory agents and modern psychoactive treatments. This review is limited by its narrative approach. Differences among TRD patient populations and experimental models may limit how broadly the findings can be applied. Also, the shortcomings of the review include limited evidence on epigenetic factors and original research which can directly link the interplay of the pathways involved in TRD. Finally, gaps in translating preclinical findings to clinical settings highlight the need for further research to confirm the proposed mechanisms.

Future perspectives must look into integration of multi-omics and longitudinal studies as essential steps to elucidate and understand the mechanism of genetic, epigenetic, and metabolic alterations interact over the course of TRD. Incorporating biomarkers that are derived from stress hormones, immune markers, and kynurenine metabolites could help establish predictive models for treatment response. Additionally, combining pharmacological and non-pharmacological interventions, such as neuromodulation, psychotherapeutic approaches, and lifestyle-based interventions will provide additional synergistic benefits in the area of research. Research into epigenetic-targeted therapies, microbiota–gut–brain interactions, and personalized medicine frameworks will be crucial to developing precision treatments for TRD. Future research should also aim to bridge preclinical findings with clinical outcomes to advance mechanistic insights and improve treatment efficacy for TRD patients. Further, exploring epigenetic-targeted therapies and metabolic interventions could lead to more personalized treatment strategies, ultimately improving therapeutic outcomes.

## Data Availability

No datasets were generated or analysed during the current study.

## Abbreviations

3-HAA: 3-Hydroxyanthranilic acid
3-HAO: 3-Hydroxyanthranilate 3,4-dioxygenase
3-HK: 3-Hydroxykynurenine
ACTH: Adrenocorticotropic hormone
ACMSD: Aminocarboxymuconate-semialdehyde decarboxylase
BBB: Blood-brain barrier
CNS: Central nervous system
CRH: Corticotropin-releasing hormone
ECT: Electroconvulsive therapy
GSRD: European group for the study of resistant depression
FDA: Food and drug administration
GR: Glucocorticoid receptor
HPA: Hypothalamic-pituitary-adrenal axis
IDO: Indoleamine 2,3-dioxygenase
IL-1β: Interleukin-1 beta
IL-6: Interleukin-6
iTBS: Intermittent theta burst stimulation
KAT: Kynurenine aminotransferase
KMO: Kynurenine monooxygenase
KA: Kynurenic acid
MDD: Major depressive disorder
NMDA: N-methyl-D-aspartate
NSSI: Non-suicidal self-injury
P-gp: P-glycoprotein
QUIN: Quinolinic acid
rTMS: Repetitive transcranial magnetic stimulation
SSRI: Selective serotonin reuptake inhibitor
TDO2: Tryptophan 2,3-dioxygenase
TRD: Treatment-resistant depression
TWAS: Transcriptome-wide association study
VNS: Vagus nerve stimulation
